# Topical Micro-Emulsion of 5-Fluorouracil by a Twin Screw Processor-Based Novel Continuous Manufacturing Process for the Treatment of Skin Cancer: Preparation and In Vitro and In Vivo Evaluations

**DOI:** 10.3390/pharmaceutics15092175

**Published:** 2023-08-22

**Authors:** Ajinkya Nitin Nikam, Angela Jacob, Ruchira Raychaudhuri, Gasper Fernandes, Abhijeet Pandey, Vinay Rao, Sheikh F. Ahmad, Ananth S. Pannala, Srinivas Mutalik

**Affiliations:** 1 Department of Pharmaceutics, Manipal College of Pharmaceutical Sciences, Manipal Academy of Higher Education, Manipal 576104, Karnataka State, India; ajinkya.nikam7@gmail.com (A.N.N.); jacobangela22@gmail.com (A.J.); ruchira1995@gmail.com (R.R.); fernandesgasper16@gmail.com (G.F.); abhijeet_pandey87@yahoo.com (A.P.); 2 STEERLife India Private Limited, No. 290, 4th Main Road, Ganapathy Nagar, Phase 3, Peenya Industrial Area, Peenya, Bangalore 560058, Karnataka State, India; vinay.rao@steerlife.com; 3 Department of Pharmacology and Toxicology, College of Pharmacy, King Saud University, Riyadh 11451, Saudi Arabia; fashaikh@ksu.edu.sa; 4 Biomaterials and Drug Delivery Research Group, School of Applied Sciences, University of Brighton, Brighton BN2 4GJ, UK; a.s.v.pannala@brighton.ac.uk

**Keywords:** 5-Fluorouracil, microemulsion, twin-screw processor, continuous manufacturing, skin cancer, squamous cell carcinoma (SCC)

## Abstract

5-Fluorouracil (5-FU), a BCS class III drug, has low oral bioavailability and is cytotoxic in nature causing severe systemic side effects when administered through the intravenous route. Topical drug delivery could potentially mitigate the systemic side-effects. Microemulsions (MEs) would be an apt solution due to enhanced partitioning of the drug to the skin. However, conventional methods for preparing MEs are inefficient since they are not continuous and are very tedious and time-consuming processes hence revealing the need for the development of continuous manufacturing technology. In our study, 5-FU MEs were prepared using a continuous manufacturing Twin Screw Process (TSP) and its efficiency in the treatment of skin cancer was evaluated. Water-in-oil MEs were prepared using isopropyl myristate as the oil phase and Aerosol OT and Tween 80 as the surfactants. The average particle size was observed to be 178 nm. Transmission electron microscopy was employed to confirm the size and shape of the MEs. FTIR study proved no physical or chemical interaction between the excipients and the drug. In vitro drug release using vertical diffusion cells and ex vivo skin permeation studies showed that the drug was released sustainably and permeated across the skin, respectively. In in vitro cytotoxicity studies, 5-FU MEs were accessed in HaCat and A431 cell lines to determine percentage cell viability and IC50. Skin irritation and histopathological examination implied that the 5-FU MEs did not cause any significant irritation to the skin. In vivo pharmacodynamics studies in rats suggested that the optimised formulation was effective in treating squamous cell carcinoma (SCC). Therefore, 5-FU MEs efficiently overcame the various drawbacks faced during oral and intravenous drug delivery. Also, TSP proved to be a technique that overcomes the various problems associated with the conventional methods of preparing MEs.

## 1. Introduction

Skin cancers are the most frequent malignancies in humans, mainly caused by cutaneous epithelial cells [1]. Skin cancers other than melanoma make up the majority of cases. Non-melanoma skin cancers that are malignant are produced by keratinised epithelial cells. These cancers include squamous cell carcinoma (SCC) and basal cell carcinoma (BCC). Even though melanoma accounts for only about 2% of all malignant skin cancers, it causes the majority of fatalities [2]. Melanoma occurs when melanocytes begin to grow uncontrollably [3]. Collagenosis, uneven pigmentation, skin wrinkling, solar keratosis in skin regions exposed to the sun, and skin telangiectasia are all common symptoms of skin cancer resulting from chronic sun damage [4].

Topical chemotherapy, excisional surgery, photodynamic therapy (PDT), radiation therapy, targeted therapy, cryotherapy, and immune response modifiers are all approaches for skin cancer treatment [5]. Each treatment has its advantages and disadvantages. Due to the intense inflammation, pain, and cosmetically undesirable scars of surgical therapies, topical therapy regimens are preferable over surgical methods [1,6,7]. Topical drug delivery offers significant advantages like enhanced bioavailability, consistent drug concentration in plasma, prolonged activity reducing dosing frequency, fewer adverse effects, and improved treatment maintenance. It also avoids first-pass metabolism and risks of intravenous administration, considering factors affecting absorption. However, drawbacks include skin irritability, allergic reactions, poor skin permeability for some drugs, difficulty absorbing drugs with big particles, and contact dermatitis causing skin inflammation [8,9].

5-Fluorouracil (5-FU) is an anti-cancer drug used to treat skin conditions like cutaneous malignant melanoma, premalignant lesions, psoriasis, SCC and BCC [10]. It inhibits thymidylate synthetase, resulting in low amounts of thymidine, which is essential for DNA synthesis, inhibiting the abnormal cell’s DNA synthesis. It is a BCS class III drug with poor permeability and good aqueous solubility. Its medicinal application is limited by less per-oral bioavailability, hydrophilicity, inconsistent metabolic rate, severe toxic effects, brief half-life, and quick clearance after intravenous injection. In our opinion, the topical route of administration of 5-FU for treating skin cancer is still the best option. Creams and solutions are among several 5-FU topical preparations for non-melanoma skin cancer treatment. Daily medications are linked with systemic and local side effects, making them less patient-friendly. Due to the lack of success in therapy and the inability to tolerate the treatment plan, a significant portion of the patients experience the development of a new issue within a specific timeframe. As a result, 5-FU’s systemic absorption must be low enough to render its topical version ineffective and harmless. The antitumor drug must be in an appropriately planned formulation that allows it to reach adequate concentrations in the epidermal layers where the tumour is situated without being absorbed into the bloodstream, making it more patient-friendly. Microemulsions (MEs) would be an appropriate solution due to enhanced drug partitioning to the skin [1,11,12,13].

The MEs are a dispersion of co-surfactants, surfactants, oil, and an aqueous phase that form a single, thermodynamically stable, and optically isotropic liquid solution with droplet diameters typically in the submicron range (less than 100 nm) [14]. Because of their tiny size, they display innovative and unusual biological or physical activity. They can offer better permeation, sustained drug release, and reduced irritation. They are thermodynamically stable across various temperatures and, unlike other emulsion systems in which the oil droplets might gradually coalesce, phase separation does not occur in the formation of MEs [15].

Oil-in-water (O/W) MEs and water-in-oil (W/O) MEs are the two most common forms of microemulsion systems. The W/O MEs may prove to be a particularly effective method of improving the percutaneous permeation of the hydrophilic drug 5-FU since they can enhance the interaction among the stratum corneum and hydrophilic drugs, alter the shape of the bilayer membrane, limit its dimensions, and easily pass through the skin’s hair follicle openings. Recently, interest in using the water/Aerosol-OT or Bis(2-ethylhexyl) sulfosuccinate sodium salt (AOT)/isopropyl myristate (IPM) ME platform for topical drug administration has increased. AOT is an anionic surfactant that can disperse a substantial volume of water in oil before creating a thermodynamically stable microemulsion. It has an excellent hydrophilic–lipophilic balance (HLB = 10.5) [16]. Another method for increasing solubilisation and creating a stable ME system is to add a co-surfactant like Tween 80, as this co-surfactant reduces interfacial tension, increases interfacial fluidity and flexibility, and prevents the development of stiff structures such as liquid crystals [10].

**Figure 1:**
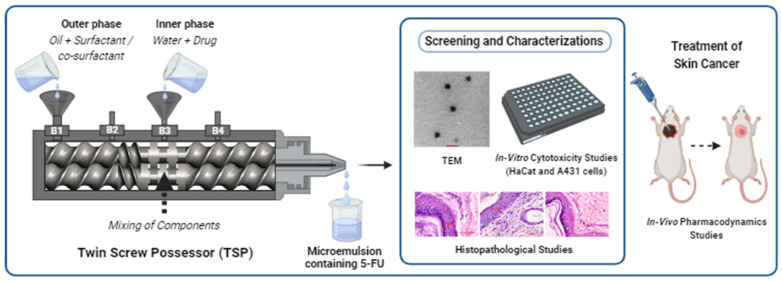
Schematic Representation of the Co-rotating Twin Screw Processor for Continuous Manufacturing of Topical Micro-emulsion of 5-Fluorouracil for Treatment of Skin Cancer.

In the studies carried out so far, MEs have been prepared by conventional methods such as magnetic stirring, homogenising, and ultrasonication which are not very efficient, not continuous, and are time-consuming. This also increases the production cost, and since batch processing is involved, this might lead to variability in the quality of the final product. Scaling up of the process from lab scale to a commercial scale is also difficult. Therefore, introducing a continuous manufacturing process like the Twin Screw Processing (TSP) technique in the production of MEs will overcome these problems and improve their potential in the pharmaceutical sector. TSP is a continuous manufacturing process that offers various advantages, including increased production capacity, lower production costs, faster product delivery, decreased space and workforce requirements, avoidance of scaling-up issues, and enhanced product quality. Because TSP is a continuous process, scaling up of the process is easier than that of a batch process because raising the batch size requires simply a longer run time with the process parameters on the same equipment. Also, from a quality point of view, continuous manufacturing has been reported to provide homogeneous quality along with low Out-of-Specification and deviation. [17,18,19,20]. In this study, a microemulsion containing 5-FU (5-FU@ME) was prepared using the TSP technique and evaluated for various parameters as illustrated in Figure 1.

## 2. Methodology

### 2.1. Materials

5-Fluorouracil, Aerosol-OT, Isopropyl myristate and Trifluoroacetic acid were all purchased from TCI Chemicals, India. Tween 80 was obtained from Loba Chemie Pvt. Ltd., Maharashtra, India. All additional chemicals utilised were of analytical grades.

### 2.2. Methods

#### 2.2.1. Preparation of 5-FU@ME Using TSP

**Figure 2:**
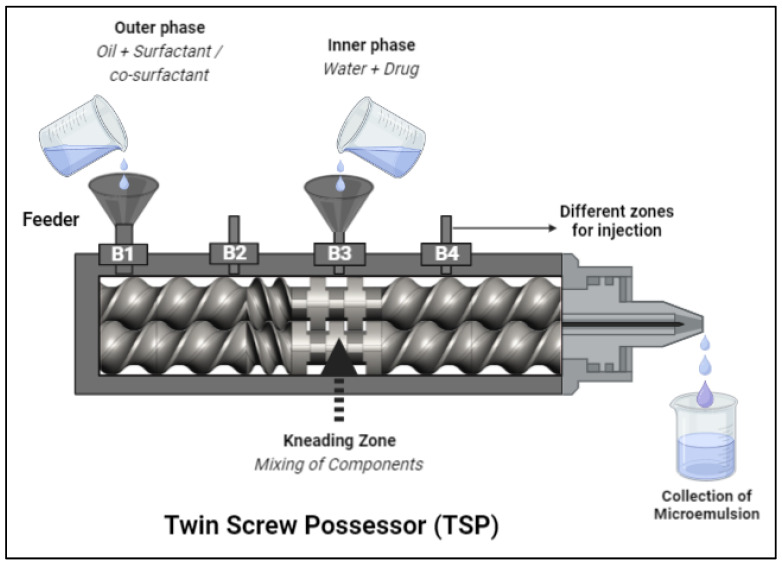
Formulation of Microemulsion using Twin Screw Processor (TSP).

A TSP (Omicron 10P, Steer Engineering, Bangalore, India) with a screw external width to internal width proportion of 1.71 was used to fabricate the 5-FU@ME. The One-Factor-at-a-Time (OFAT) method was utilised to optimise the 5-FU@ME; it involved varying one factor at a time while holding the others constant. The outer phase (oil) along with surfactant and co-surfactant (pre-mixed into oil) was introduced into the feeder and an inner phase (water + drug) was introduced from the kneading zone (B3) of TSP thereafter. The microemulsion was collected from the outlet as shown in Figure 2. The process was optimised by varying rotation speed of the screws. The impact of this parameter on the quality of the MEs (size, zeta potential, polydispersity index) was systematically studied.

#### 2.2.2. Droplet Size, Polydispersity Index and Zeta Potential

Utilising a particle size analyser (NanoZS, Malvern Instruments, Malvern, UK), the droplet size, polydispersity index and zeta potential of the developed 5-FU@ME formulations were assessed using electrophoretic light-scattering techniques and dynamic light scattering. Triplicates were taken for all the measurements [1,12,21,22].

#### 2.2.3. Thermodynamic Stability Studies

The formulation’s physical stability was checked based on the following criteria:

Heating-cooling cycle (6 cycles at temperatures of 4 °C and 45 °C) was used to assess the stability of MEs (packed in Tarsons Cliklok 2.0 mL Micro Centrifuge Tube sealed with parafilm) with respect to phase separation. The MEs were kept for 48 h at each temperature. Centrifugation test was performed to assess the phase separation of MEs by centrifuging them for at 3500 rpm for 30 min to determine the phase separation. In Freeze–Thaw test, the MEs were observed for phase separation at temperatures of −21 °C and +25 °C for a period of 48 h and 3 cycles at a single temperature [1,10,15,21].

#### 2.2.4. Fourier Transform Infrared Spectroscopy (FTIR)

To examine the interactions among the excipients and drug, the FTIR analysis of 5-FU, isopropyl myristate, AOT, Tween 80, and the optimised formulation was performed using a BRUKER-ALPHA II ATR-FTIR (Bruker, Heidelberg, Germany) spectrophotometer. Further analysis of the spectra was carried out for the functional groups to determine the drug–excipient interaction [23,24,25,26].

#### 2.2.5. Transmission Electron Microscopy (TEM)

TEM (TEM; FEI, Tecnai G2 Spirit Bio-Twin, Eindhoven, The Netherlands) was employed to conduct the morphological and structural examination of 5-FU MEs. The machine was operated at 100 kV at magnification of 40,000× to 60,000×. The 5-FU@ME was put in the form of a drop on a copper grid, and a filter paper was utilised to eliminate the surplus. Subsequently, a high-resolution transmission electron microscope (TEM) was used to examine the grid to determine the 5-FU@MEs morphology [1,21].

#### 2.2.6. Rheological and pH Measurements

The viscosity of the MEs was measured utilising Brookfield Viscometer (BDV-II+ Pro, Model: D 220, Brookfield, Middleboro, MA, USA) at shear rates of 50 and 100 rpm using a torque of 0.8%. Spindle F-96 (T-Bar) was used for analysis [1,12]. pH of the microemulsions was measured using a digital pH meter (μ pH System 361, Systronics, Ahmedabad, India).

#### 2.2.7. Estimation of 5-FU in 5-FU@MEs Using HPLC

A previously reported HPLC method, with few modifications, was used to quantify 5-FU in the samples [27]. A UV-visible detector, an auto sampler, and dual piston pumps were integrated in the HPLC system (Shimadzu, Kyoto, Japan). Chromatograms were analysed using LC solution software (version 5.57). Using a reverse phase Luna C18 column (Phenomenex, Hyderabad, India) chromatographic separation was carried out. Acetonitrile and aqueous phase (containing water balanced to pH 3.0 using Trifluoroacetic acid) in a proportion of 02:98 *v/v* was used at a flow rate of 0.8 mL/min. With a 15 min run time, the analysis was performed at 265 nm. With a r^2^ value of 0.999, the calibration curve (Figure S1; Supplementary Information) for the analytical specimens displayed excellent linearity in the concentration range of 2 to 16 μg/mL.

#### 2.2.8. Determination of Drug Content

Drug content in the formulation was measured by extracting 5-FU from 1 mL of the formulation into 10 mL ethanol and analysed using HPLC as described previously [12,28].

#### 2.2.9. In Vitro Drug Release Study

In vitro drug release experiment was conducted utilising vertical Franz diffusion cells. Cellulose Dialysis membrane (Dialysis Membrane-110, HiMedia) was arranged between the receptor and donor compartments. Phosphate buffer saline (3.5 mL; PBS) pH 7.4 was transferred to the receptor compartment of each cell with continuous stirring. One mL of the drug solution or formulation (equivalent to 5 mg of drug) was transferred to the donor section. The samples (350 μL) were withdrawn from the receptor compartment through the sampling port and the same amount of PBS pH 7.4 was used for replacing it. The samples were analysed for 5-FU using HPLC as described above [28].

#### 2.2.10. Ex Vivo Skin Permeation Studies

The animal experiment protocol was permitted by Institutional Animal Ethics Committee, Kasturba Medical College, Manipal (IAEC No.: IAEC/KMC/96/2021). The ex vivo skin permeation experiment was performed on fully layered Wistar rat skin. Combination of Xylazine 30–40 mg/kg + Ketamine 300–360 mg/kg was injected intraperitoneally to sacrifice the rats. After removal of dorsal hair, the fully layered rat’s skin was taken, and the connective tissues and fat was surgically removed and washed using normal saline. Vertical diffusion cells were employed to mount the intact skin. The stratum corneum was placed facing the donor section and the dermis side towards the receptor section. PBS (3.5 mL; pH 7.4) was transferred into the receptor compartment. One mL of the drug solution or formulation (equivalent to 5 mg of drug) was transferred to the donor compartment. Remaining procedure is same as mentioned in in vitro drug release study (Section 2.2.9) [7,28]. To find the amount of 5-FU present in the skin following the ex vivo permeation experiment, drug was extracted from the skin by following previously reported method and quantified using HPLC [7].

#### 2.2.11. In Vitro Cytotoxicity Studies

The Human Skin Keratinocytes (HaCaT) and Human Skin adenocarcinoma cell line (A431) were purchased from ATCC, USA and NCCS, Pune, India, respectively. The cells were incubated in Dulbecco’s modified Eagle’s medium (DMEM) aided with FBS (10%) and antibiotic-antimycotic (1%) solution in a CO_2_ incubator at 37 °C in an atmosphere of CO_2_ (5%), O_2_ (18–20%). The pure drug, blank ME and 5-FU@ME-2 were assessed for cell toxicity on HaCaT and A431 cell lines by MTT assay [29]. The % cell viability was determined utilising below formula [30]:

% Cell viability = Abs of treated cells/Abs of Untreated cells × 100

To calculate the IC50 value, the linear regression equation Y = Mx + C was used. M and C values were obtained from the viability graph with Y = 50 in this instance.

#### 2.2.12. Skin Irritation and Histopathological Studies

The MEs were examined for skin irritation on male Wistar rats (200–250 g) after Institutional Animal Ethics Committee approval. The rats were placed in standard laboratory conditions (25 ± 2 °C and 35–45% RH). Water was given as required and standard diets were maintained during the study. The rat’s dorsal side was shaved from tail to head with an electric clipper. The animals were placed into three groups (n = 6) and were given the following treatments:

Group I: Treated with 0.5 g of Blank formulation (no drug)
Group II: Treated with 0.5 g of Optimized formulation (5-FU@ME-2)
Group III: Treated with Formalin (0.8% *v*/*v*)

Above given formulations were applied on the back of the rats within an area of 5 cm^2^ for 7 days and the animals were monitored on a daily basis for change in morphology, skin colour and the development of erythema and oedema, and mean oedema and erythemal scores were noted down [1,10,15].

After the skin irritation study was completed, the animals were sacrificed. The area of the skin that received the treatment was subjected to histopathological study using haematoxylin and eosin (H & E) stains. The detailed procedure of H & E staining is given in Supplementary Information (Section S2) [1,11,12].

#### 2.2.13. In Vivo Pharmacodynamics Studies

The in vivo efficacy of the 5-FU@ME-2 and marketed formulation (Flonida 1% *w*/*w*) was investigated in the rats induced with SCC. The procedure for the induction of SCC in animal model was adapted from previously reported article with few modifications [31]. 7,12-Dimethyl-benzanthracene (DMBA) solution (1% *w*/*v*) was freshly prepared in acetone for the induction of SCC in male Wistar rats. Around 200 µL of this solution was topically sprayed on the shaved part of rat skin and exposed to UV-light of wavelength 311 nm for 1 min on every alternate day for a period of 3 months. After 3 months of visualisation of SCC induction, the skin was excised, stored in 10% formalin solution, and taken for histopathological examination. The skin sections were observed to confirm SCC induction. After confirming the induction of SCC in animals by histopathological examination of SCC induced skin, the grouping of the animals (n = 6) was carried out as follows.

Group 1: Normal Control (no disease induced, no drug treatment)
Group 2: Positive control (disease induced, no treatment)
Group 3: Marketed formulation (Flonida 1% *w*/*w*)
Group 4: Optimised Formulation (5-FU@ME-2)

The marketed formulation (Flonida cream; 1% *w*/*w* 5-FU) and optimised formulation (5-FU@ME-2) were applied topically on the disease-induced rat skin once daily for a period of one month to check their efficacy. The efficacy of the formulations was assessed by visual observation of SCC induced area of the skin and histopathological studies of the excised skin immediately after the treatment period.

## 3. Results and Discussion

### 3.1. Preparation of MEs Using TSP

For formulating MEs using TSP, IPM was chosen as the oil phase due to its non-greasy nature, with AOT as the surfactant, and Tween 80 as the co-surfactant. For optimisation, the OFAT approach was used. It requires retaining the other process parameters constant while changing one process parameter at a time. The weight percentage of the oil and water phases were varied for preparing different batches of the MEs before finalising the optimised formulation. The weight percentage of the oil phase was varied between 50–65% and of the water phase between 10–25% (Table 1). 5-FU@ME-2 was found to be stable and was selected as the optimised formulation. The other three batches (5-FU@ME-1, 5-FU@ME-3 and 5-FU@ME-4) were found to be unstable as the oil phase and water phase were becoming separated at room temperature, and therefore were not chosen for further studies. The outer phase (oil) along with surfactant and co-surfactant was introduced into the feeder and an inner phase (water + drug) was introduced from the B3 region of TSP thereafter. The RPM was set at 100 after determining the effect of increasing RPM on the droplet size of the MEs. At minimum screw rotation, i.e., 100 RPM, the size of the 5-FU@ME-2 droplet was observed to be 178.7 ± 3.22 nm and the polydispersity index (PDI) was found to be 0.247 ± 0.03. The size of the droplet was observed to be increasing along with an increase in RPM. At maximum screw rotation, i.e., 250 RPM, the droplet size was found to be 2795.6 ± 9.65 nm and the PDI was found to be 1.0 ± 0.00. This might be due to the formation of air bubbles during high-speed mixing in the kneading zone of the TSP. The droplet size increment with the increase in RPM is shown in Table 2. Therefore, 100 RPM was fixed for the optimised formulation of 5-FU@ME-2. The temperature of the barrels was set at room temperature. The ME was collected after mixing from the outlet and subjected to further evaluation.

### 3.2. Polydispersity Index and Droplet Size

In MEs, determination of the droplet size is critical since a smaller globule-sized emulsion leads to a larger interfacial area, which helps to improve partitioning of the drug and absorption [10]. As the electrical charges present on particles determine the flocculation rate, the zeta potential is primarily used for measuring flocculation. It must be zero or neutral to maintain the stability of MEs. The droplet size of 5-FU@ME-2 was found to be in a nano size range [1]. The mean size of particle was observed to be 178.7 ± 3.22 nm (Figure S2; Supplementary Information). The average polydispersity index was observed to be 0.247 ± 0.03 and the average zeta potential was determined to be −1.011 ± 0.21 mV, whereas the size of 5-FU-free@ME-2 was found to be 145.9 ± 7.38 nm and the PDI was found to be 0.270 ± 0.07. The low PDI value indicates the uniformity in size [1,32]. The negative zeta potential implies that the globules were free of charge, indicating that the system was stable. As a result, 5-FU@ME-2 was determined to be stable [28].

### 3.3. Thermodynamic Stability Studies

The prepared 5-FU@ME-2 showed good thermodynamic stability after passing through six cycles of heating–cooling (4 °C and 45 °C) and three cycles of the freeze–thaw test (−21 °C and +25 °C). The centrifugation test was carried out at 3500 rpm for 30 min. Centrifugation can speed up the creaming rate, demonstrating that emulsion disintegration is linked to gravitational force. The 5-FU@ME-2 did not exhibit any phase partition, change in colour, drug precipitation, creaming, or cracking, indicating its physical stability [1]. Since, 5-FU@ME-2 showed stability, it was subjected to other studies.

### 3.4. Fourier Transform Infrared Spectroscopy

**Figure 3:**
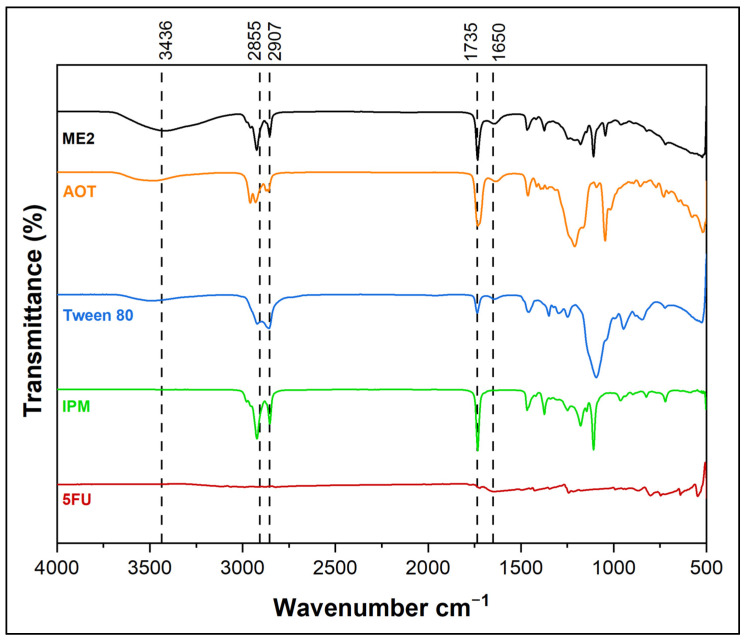
FTIR spectra of 5-FU, IPM, Tween 80, AOT, and ME2.

Interaction between the excipients and the drug is essential to understand the release of the drug from the formulation. Pure 5-FU and the excipients were examined in the 500–4000 cm^−1^ range, employing ATR-FTIR. The drug displayed peaks due to the secondary amino group as a broad band between 3000 and 3500 cm^−1^, the cyclic ketonic group around 1650 cm^−1^, the C–F stretching at 1275 cm^−1^, and the C=C group [23,24,25,26]. Tween 80 showed symmetric and asymmetric stretching bands at 1735 cm^−1^ due to the C=O ester group, (–CH2) at 2907 and 2855 cm^−1^, respectively, and a proximate band at 3436 cm^−1^ which depicted the hydroxyl stretching vibration [33]. A C–O stretching band could be seen at 1045 cm^−1^ for AOT. O–H and C=O stretching modes could be seen at 2940 cm^−1^ and 1740 cm^−1^, respectively, for IPM (Figure 3). All the distinctive peaks of the excipients and drug were also observed in the spectra obtained for the formulation (5-FU@ME-2) (Figure 3), proving that there were no physical or chemical reactions amongst the drug and the excipients and that all of them were stable in the formulation.

### 3.5. Transmission Electron Microscopy

**Figure 4:**
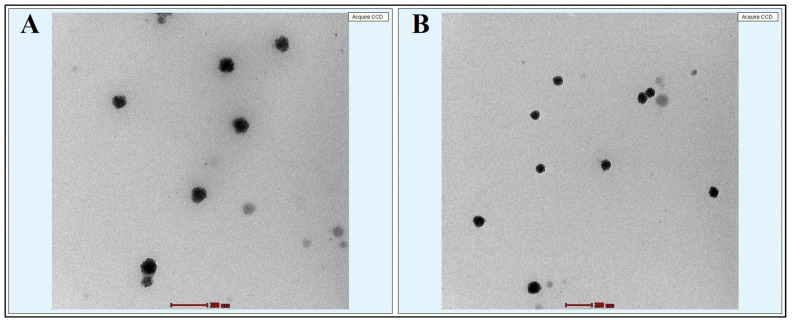
TEM analysis of MEs with 200 nm scale ((**A**): 5-FU-free@ME-2; (**B**) 5-FU@ME-2).

Transmission electron microscopy (TEM) was carried out to define the size and shape of the prepared 5-FU-free@ME-2 and 5-FU@ME-2. Furthermore, the droplet size of 5-FU@ME-2 was larger than that of 5-FU-free@ME-2, which was in accordance with the previously reported work [34]. Both the results showed the *w*/*o* MEs globules as scattered black spherical spots on a light backdrop, with diameters < 100 nm (Figure 4). The globules were found as discrete entities and did not show any aggregation. Solubility of MEs is affected by particle size; as particle size reduces, solubility increases. Therefore, the TEM picture validates the size, spherical shape, and equivalent distribution of 5-FU@ME-2.

### 3.6. Rheology, pH and Drug Content

The viscosity of 5-FU@ME-2 was recorded using a Brookfield viscometer. At a spindle speed of 50 RPM, viscosity was found to be 16.8 cP and torque was 0.8% and at a spindle speed of 100 RPM viscosity was found to be 9.6 cP and torque was 0.8%. The viscosity was found to decrease with an increase in shear rate. The shift in shape of the globules to non-spherical from spherical may be responsible for the reduction in viscosity. The low viscosity indicates a weaker system structure that allows for easier application on the skin [1]. The pH of 5-FU@ME-2 was found to be 6.20 ± 0.01, which is ideal for application to the skin [1]. Drug content was observed to be between 96.6% to 98.59%, which suggests that the drug was evenly dispersed across the formulation and that loss of the drug during the formulation of 5-FU@ME-2 was minimal [21].

### 3.7. In Vitro Drug Release Study

**Figure 5:**
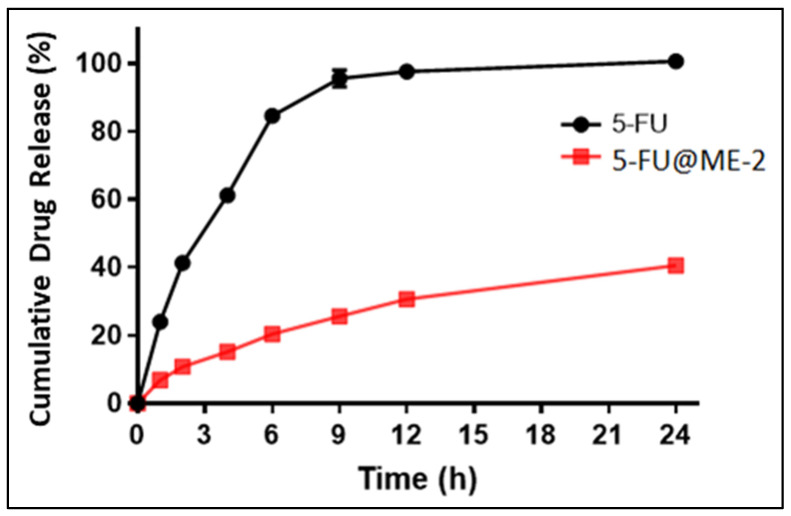
In vitro drug release profile of 5-FU across cellulose membrane.

An in vitro drug release study was carried out utilising cellulose membrane in PBS pH 7.4. It demonstrated that 5-FU@ME-2 in comparison to the standard drug solution (5-FU) showed sustained drug release properties due to the oil phase’s resistance to drug diffusion into the release medium from the inner phase [13]. The percentage cumulative drug release (% CDR) in 24 h in PBS pH 7.4 from the 5-FU@ME-2 was found to be 40% and from the standard drug solution (5-FU) it was almost 100% (Figure 5), and this is in accordance with the previously reported article [35].

### 3.8. Ex Vivo Skin Permeation Studies

**Figure 6:**
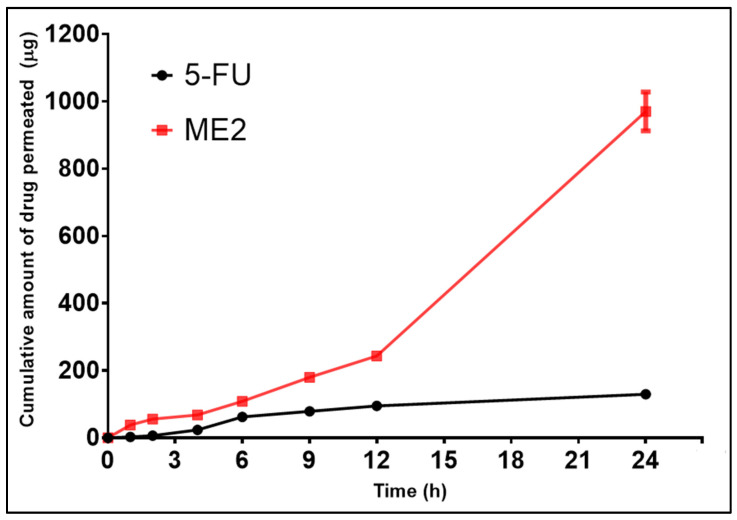
Ex vivo permeation profile of 5-FU across rat skin.

The ex vivo skin permeation study was carried out by using full-thickness rat skin in PBS 7.4 pH buffer. The cumulative drug permeation (CDP) at 24 h from 5-FU@ME-2 was found to be 940.93 ± 5.53 µg and from the standard drug solution (5-FU), it was found to be 132.93 ± 5.11 µg (Figure 6) [1,7].

The ability of microemulsion to increase the permeation of 5-FU through the skin can be attributed to several compelling mechanisms, acting independently or in conjunction. Since it is W/O microemulsion, the lipophilic nature might be facilitating migration of 5-FU into the stratum corneum layer that is extremely lipidic. Through interactions with lipid and/or protein complexes, the W/O microemulsion might be accountable for facilitating 5-FU penetration through the highly organised, dense structure of viable cells within the skin. Another reason for enhanced permeation might be due to smaller particle size, as there will be a maximised surface area of the particles and an increased solubility pressure of the particles.

The curve’s linear portion was used to determine the flux. Nearly linear permeation curves demonstrate the stratum corneum and epidermal layer’s preserved integrity. Flux of the standard drug solution was much less (5.63 ± 0.11 µg/cm^2^/h) than that of 5-FU@ME-2 (19.63 ± 0.12 µg/cm^2^/h) thus indicating enhanced absorption of 5-FU from the 5-FU@ME-2 (Table 3) [1].

The therapeutic effectiveness of 5-FU in skin cancer treatment will be limited if its concentration in the skin is insufficient. Longer action is ensured by a greater amount of drug at the target location [1]. In PBS pH 7.4 the retention of 5-FU from 5-FU@ME-2 was found to be 58.32 ± 0.09 µg/cm^2^ in the skin compared to the standard drug solution which was 294.46 ± 1.12 µg/cm^2^ (Table 3). This proved that there was a greater accumulation of the drug in the target site in the case of 5-FU@ME-2 which helps create greater therapeutic efficiency by providing prolonged release of the drug. This will also reduce the dosing frequency required [15].

### 3.9. In Vitro Cytotoxicity Studies

**Figure 7:**
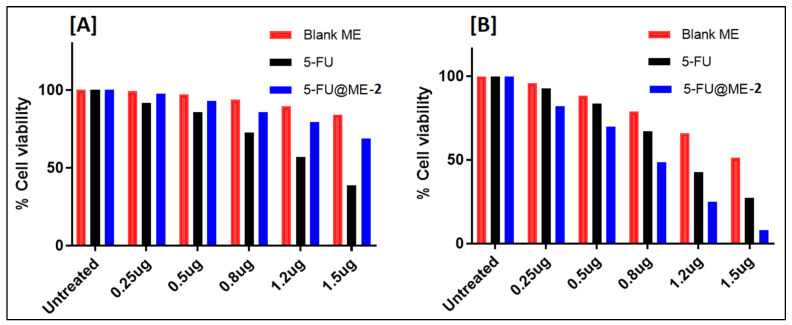
The graph shows the % cell viability values observed with pure drug, blank ME, and 5-FU@ME-2 against HaCat cells (**A**) and A431 cells (**B**) in MTT assay.

Cytotoxicity, in terms of % cell viability, of pure drug, blank ME, and 5-FU@ME-2 was evaluated against HaCat and A431 cells (Figure 7A,B). With an increase in the drug concentration in the HaCat cells (Figure 7A), the cell viability decreased in the case of the pure drug solution; however, in the case of 5-FU@ME-2, the decrease in cell viability was much less. 5-FU@ME-2 had less cytotoxic effect, although not considerably different, on the HaCat cells compared with plain 5FU. It may be because microemulsion tends to protect 5-FU from immediate release and metabolism. This indicated the safety of the developed formulation to normal cells and the fact that the formulation protects the drug to reduce its toxicity. On the contrary, in A431 cells (Figure 7B), the formulation 5-FU@ME-2 showed greater cytotoxicity as compared to the pure drug solution; this may be because the microemulsion might have increased the cell permeability and uptake of the 5-FU into the A431 cells. Similar observations have been reported previously in the literature for anti-cancer drugs and formulations, where formulations showed more cytotoxicity to cancer cells and less cytotoxicity to healthy cells [36,37,38,39,40]. The IC_50_ values of blank ME, pure drug and 5-FU@ME-2 are listed in Table 4. In HaCat cells, the IC_50_ value of plain 5-FU was found to be 1.3 µg/mL, which is less than the IC_50_ value in the 5-FU@ME-2 (2.42 µg/mL). In A431 cells, 5-FU@ME-2 was found to be more cytotoxic (IC_50_: 0.79 µg/mL) compared to the plain drug (IC_50_: 1.08 µg/mL). On the other hand, blank ME demonstrated less cytotoxicity in both the cell lines as compared to the pure drug (5-FU) and 5-FU@ME-2 indicating that the components used in the preparation of ME are safe and non-toxic. Further detailed studies are required to confirm these results.

### 3.10. Skin Irritation Studies

Compared to the control group, the application of 5-FU@ME2 caused no irritation in the skin of the rats, as shown in Table 5. Because no skin abnormalities were seen, the resultant primary irritation index values were found to be within safe and acceptable levels. Skin tolerance for topically applied formulation components is also shown by blank formulation scores. The typical irritant, formalin (0.8% *v/v*), caused significant cutaneous redness, erythema, and oedema. 5-FU@ME-2 was found to be safe for the effective treatment of skin cancer [10,21].

**Figure 8:**
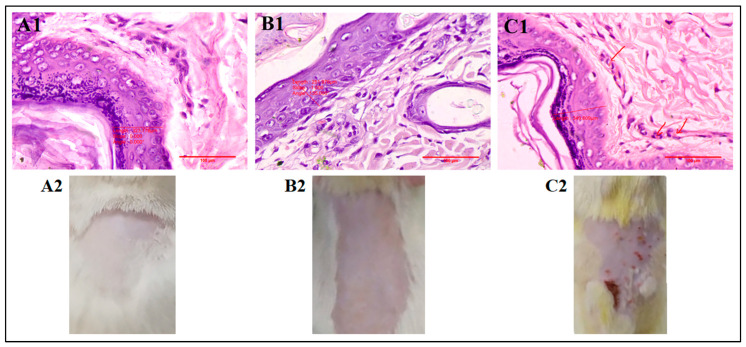
Microscopic images and photographs of rat skin in histopathological studies ((**A1**,**A2**): Blank MEs; (**B1**,**B2**): 5-FU@ME-2; (**C1**,**C2**): 0.8% Formalin) (red arrows indicate infiltration of inflammatory cells).

In histopathological studies, skin morphology did not alter significantly because of any of the formulations (blank ME and 5-FU@ME-2) (as seen in Figure 8). The upper epidermal layer’s keratin coverage seemed thin and peeled away. Although the size of the cells appeared to have grown, which is reversible, the epidermal cells did not show any increase in the number, which may occur due to inflammation. Compared to the formulation (5-FU@ME-2) and blank ME treated groups, the epidermal layer in the formalin treated group had substantial damage with infiltration of inflammatory cells (Figure 8). Therefore 5-FU@ME-2 was found to be safe on the skin as it did not show any anatomical or pathological changes [1,11,21].

### 3.11. In Vivo Pharmacodynamic Studies

**Figure 9:**
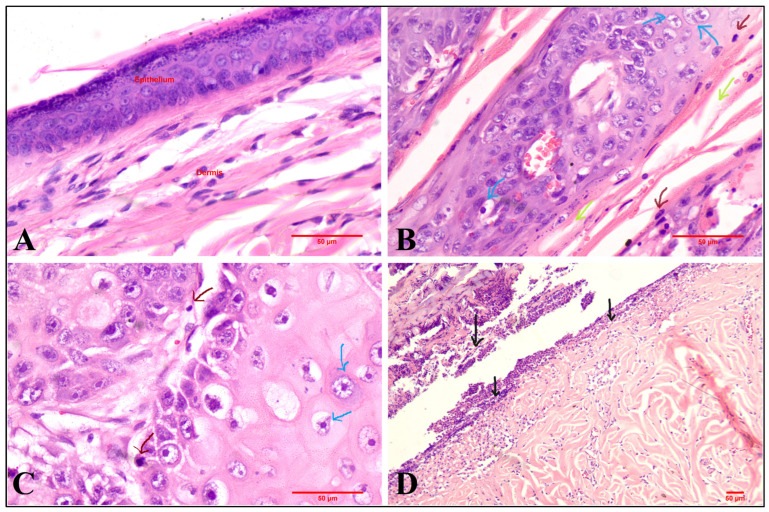
Photomicrographs of histopathological evaluation of rat skin stained with H and E in in vivo pharmacodynamics study ((**A**): Negative control; (**B**): Positive control; (**C**): Marketed formulation; (**D**): Optimised Formulation [5-FU@ME-2]) (Brown arrow: Mitotic figures; Blue arrow: Epithelial Island; Green: Keratinisation; Black arrow: Ulcerative epithelium).

The induction of SCC was confirmed by histopathological examination. The slides showed ulcerative epithelium with areas of hyperkeratosis (Figure 9B). Epithelial islands were seen invading the connective tissue. The epithelial cells showed basilar hyperplasia, nuclear pleomorphism, dyskeratosis and many mitotic figures. The epithelial islands revealed keratin pearls, with few areas also showing abscess formation. Mild infiltration of inflammatory cells like lymphocytes and eosinophils was seen in connective tissue. Four to five per HPF (High power field) mitotic figures were seen, concluding in the confirmation of squamous cell proliferations in the tissue.

**Figure 10:**
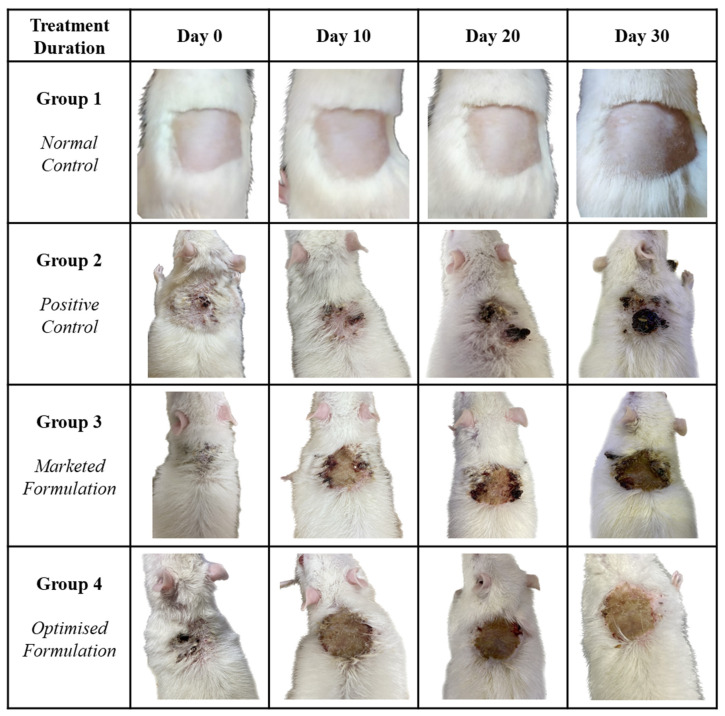
Visual observation of Group 2 (Positive control), Group 3 (Marketed formulation), and Group 4 (Optimised Formulation).

During one month of the treatment, all the groups were visually observed for the whole treatment period. In comparison to the positive group (Group 2; disease induced, no treatment), considerable reduction in the SCC area was observed in the treatment group of 5-FU@ME-2 (Group 4), whereas marketed formulation (Group 3) showed a significantly slow treatment regime as shown in Figure 10. The results clearly indicate the improved pharmacodynamics profile of the drug when incorporated into ME, typically due to the nature of 5-FU@ME-2 which is a lipidic nanosystem. These types of ME carrier systems have been found advantageous as they quickly integrate into the tumour tissues due to higher occlusiveness prompted by their lipidic nature and large surface area provided by their nanosize [41,42].

The skin tissue from all the groups was excised and observed for histopathological changes occurring in the treatment groups as compared to the positive control group. Squamous cell proliferation in the positive group tissue (Group 2; disease induced, no treatment), was validated as discussed before and depicted in Figure 9B. These observations were then compared with all the other treatment groups by counting 10 mitotic figures from each group in 10 HPF and the results obtained are given in Table 6. Group 3 (marketed formulation) showed ulcerative epithelium, with epithelial islands invading the connective tissue. Mild infiltration of inflammatory cells like lymphocytes and eosinophils was seen in the connective tissue. Some areas showed 1–2 mitotic figures HPF, indicating a slow treatment regime of the marketed formulation which was also confirmed by visual observation. In Group 4 (5-FU@ME-2), the ulcers observed in the epithelium were decreased, whereas there were no epithelial islands in the connective tissue. The dermis showed bundles of collagen with little acute and chronic inflammatory infiltration and the lower dermis was edematous with mixed inflammatory infiltration. There were no carcinomatous areas found in the tissue, leading to the conclusion that the formulation is effective in treating SCC.

The therapeutic effectiveness of topically applied molecules is determined by their retention within the skin and systemic absorption at the targeted region. Increased local drug levels will aid in more aggressive disease targeting without causing systemic ad-verse effects. As a result, a good topical medication delivery method may readily solve these problems. MEs can improve drug retention in the skin, resulting in fewer dose intervals, less adverse effects, and shorter treatment regimes. 5-FU@ME-2 MEs were successfully formulated using the TSP technique, which is being reported for the first time. The optimised formulations showed sustained drug release, significant permeation coefficient values and enhanced skin retention which thereby help in having greater therapeutic efficiency. Optimised ME formulation was less toxic towards HaCat cell lines and more toxic towards A431 cancer cell lines. Skin irritancy and histopathology investigations established the safety of 5-FU@ME-2. When optimised formulation was subjected to in vivo pharmacodynamic studies, there were no/negligible carcinomatous areas found in the SCC-induced animal tissue, leading to the conclusion that the formulation is effective in treating SCC. 5-FU microemulsion can help patient compliance by reducing treatment duration and eliminating the negative effects of conventional medications. Furthermore, TSP was proven to be a very efficient technique for the formulation of water-in-oil microemulsions. This continuous manufacturing technique can overcome the drawbacks faced during the conventional methods of preparation of microemulsions such as requirement of more time, non-continuous nature, batch processes, higher production cost and variability in quality of the final product.

## 4. Conclusions

In conclusion, the present study explains the successful development of an optimised 5-FU@ME-2 microemulsion formulation using the novel TSP technique. The microemulsions demonstrated improved therapeutic efficacy in treating skin cancer. This advancement holds great promise for topical medication delivery, allowing for hostile disease targeting while minimising systemic adverse effects. The formulation’s safety profile was established through cytotoxicity, skin irritancy, and histopathology investigations. In vivo pharmacodynamic studies further validated its effectiveness in treating SCC, showing reduced carcinomatous areas. The use of 5-FU microemulsion not only enhances treatment outcomes but also enhances patient compliance by reducing treatment duration and minimising the side effects of conventional medication. Moreover, the innovative TSP technique offers a continuous manufacturing approach for water-in-oil microemulsions, addressing the limitations of traditional methods and paving the way for future research and development in this field. However, detailed cell line, preclinical in vivo, and stability studies are required to confirm the results of the present study.

## Figures and Tables

**Table 1:** Formulation trials for 5-FU@ME.

Batch Code	Percent Quantity of Each Component					
	Inner Phase		Outer Phase			Total
	5-FU	Water	AOT	IPM	Tween 80	
5-FU@ME-1	0.50	25	18.5	50	6	100.00
5-FU@ME-2	0.50	20	18.5	55	6	100.00
5-FU@ME-3	0.50	15	18.5	60	6	100.00
5-FU@ME-4	0.50	10	18.5	65	6	100.00

**Table 2:** Droplet size, PDI and zeta potential data of 5-FU@ME-2 with increase in RPM of TSP.

RPM in TSP	Droplet Size (nm)	PDI	Zeta Potential (mV)
100 RPM	178.7 ± 3.22	0.247 ± 0.03	−1.011 ± 0.21
150 RPM	978.1 ± 5.56	1.000 ± 0.00	−18.05 ± 1.09
200 RPM	1601.6 ± 8.76	0.977 ± 0.08	−4.65 ± 0.34
250 RPM	2795.6 ± 9.65	1.000 ± 0.00	−8.54 ± 0.92

All values are presented as Mean ± SD, n = 3.

**Table 3:** Ex vivo skin permeation parameters of 5-FU@ME-2.

Systems	Flux (µg/cm^2^/h)	Q_24_ (µg)	Drug Content in Skin (µg/cm^2^)
5-FU solution	5.63 ± 0.11	132.93 ± 5.11	58.32 ± 0.09
5-FU@ME2	19.63 ± 0.12	940.93 ± 5.53	294.46 ± 1.12

All values are presented as Mean ± SD, n = 3; Q_24_ = Cumulative amount of drug permeated at the end of 24 h.

**Table 4:** The table shows the IC_50_ concentrations of the Pure Drug, Blank ME, and 5-FU@ME-2 against HaCat and A431 cell lines after the incubation period of 24 h.

Sr. No.	Sample Code	IC_50_ (µg/mL)	
		HaCat	A431
1	Blank ME	4.5	1.59
2	Pure Drug	1.3	1.08
3	5-FU@ME-2	2.42	0.79

**Table 5:** Primary skin irritation studies of 5-FU@ME-2 and blank ME.

Formulations	Reaction Grade Observed		Primary Irritation Index
	Erythema	Oedema	
Positive Control	2.33 ± 0.33	2.17 ± 0.31	2.25 ± 0.25
Blank ME	0.00 ± 0.00	0.00 ± 0.00	0.00 ± 0.00
5-FU@ME-2	0.10 ± 0.10	0.09 ± 0.05	0.07 ± 0.06

All values have been shown as mean ± SD, n = 6. Scale of erythema: 0 = none; 1 = slight; 2 = well defined; 3 = moderate; and 4 = scar formation. Scale of oedema: 0 = none; 1 = slight; 2 = well defined; 3 = moderate; and 4 = severe.

**Table 6:** Histopathological evaluation of treatment groups.

Treatment Groups	Degree of Keratinisation	Nuclear Pleomorphism	Mitosis	Inflammatory Infiltration
Group 1	−	−	−	−
Group 2	+++	++++	+++	+++
Group 3	+	++	++	+++
Group 4	−	−	−	++

− Nil, + mild, ++ moderate, +++ severe, ++++ very severe.

## Data Availability

The data may be available on request.

## Supplementary Materials

The following supporting information can be downloaded at: https://www.mdpi.com/article/10.3390/pharmaceutics15092175/s1, (S1. Calibration curve of 5-FU, S2. Processing of tissues for histopathology studies and S3. Polydispersity index and droplet size).
