# Supplementary material for: Topical Micro-Emulsion of 5-Fluorouracil by a Twin Screw Processor-Based Novel Continuous Manufacturing Process for the Treatment of Skin Cancer: Preparation and In Vitro and In Vivo Evaluations
Source: Pharmaceutics. 2023 Aug 22;15(9):2175. doi: 10.3390/pharmaceutics15092175 (PMC10534867; doi:10.3390/pharmaceutics15092175)
Supplement: Supplementary file 1 [file pharmaceutics-15-02175-s001.zip › pharmaceutics-2555765-supplementary.pdf]

Supplementary Information

S1. Calibration curve of 5-FU

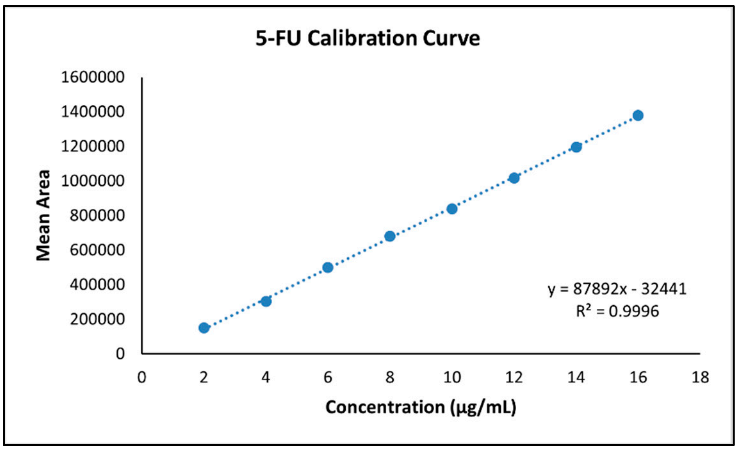

Figure S1. Calibration curve of 5-FU.

S2. Processing of tissues for histopathology studies

The processing of tissues for histopathology studies involves a meticulous series of steps to ensure accurate and detailed examination under a microscope. After the tissue was preserved in 10% formalin for 48 hours, it underwent a precise processing regimen as outlined in Table S1. The tissue was sequentially immersed in alcohol solutions of varying concentrations, gradually dehydrating it. Subsequently, the tissue was infiltrated with absolute alcohol and then xylene to facilitate the removal of water and alcohol, ultimately allowing for proper embedding in paraffin wax. This embedding process involves transferring the tissue to a mould filled with molten paraffin wax. Thin sections, approximately 4 microns thick, were carefully cut using a microtome, ensuring precision and consistency. These sections were then floated in a water bath and carefully placed onto microscopic slides.

Table S1. Processing of tissues in histopathological studies.

No	Processing solution	Time
1	70% Alcohol	30 min
2	90% Alcohol	30 min
3	Absolute Alcohol I	1 h
4	Absolute Alcohol II	1 h
5	Absolute Alcohol III	1 h
6	Xylene I	45 min
7	Xylene II	45 min
8	Paraffin Wax I	30 min
9	Paraffin Wax II	3-4 h

Table S2 outlines the detailed steps for Hematoxylin and Eosin (H&E) staining, a fundamental technique that imparts colour to cellular components aiding in microscopic analysis. After de-paraffinisation using xylene and rehydration through alcohol solutions, the

tissue was stained with Hematoxylin, highlighting nuclei. A brief rinse in acid alcohol and subsequent rinses in running water were followed by counterstaining with Eosin to visualise cytoplasmic structures. The stained sections were dehydrated again using alcohol solutions and xylene and then covered with a coverslip.

Table S2. Hematoxylin and Eosin staining in histopathological studies.

No	Staining solution	Time
1	Xylene I	5 min
2	Xylene II	5 min
3	Xylene III	10 min
4	100% Alcohol	2 min
5	70% Alcohol	2 min
6	50% Alcohol	2 min
8	Running water	2 min
8	Hematoxylin	2 min
9	Running water	1 min
10	Acid alcohol	2 dips
12	Running tap water	10 min
13	Eosin	30-60 sec
14	90% alcohol	1 dip
15	100% alcohol	2 min
16	Xylene	5 min

An LX-500 LED trinocular Research microscope (Labomed) equipped with a MiaCam CMOS AR 6pro microscope camera connected to image AR pro software was employed to visualise and document the prepared slides.

S3. Polydispersity index and droplet size

Figure S2 shows the representative particle distribution curves of 5-FU@ME-2 droplets and polydispersity index (PDI). These results offer valuable insights into the distribution of particle sizes within the sample and the overall size characteristics of the droplets.

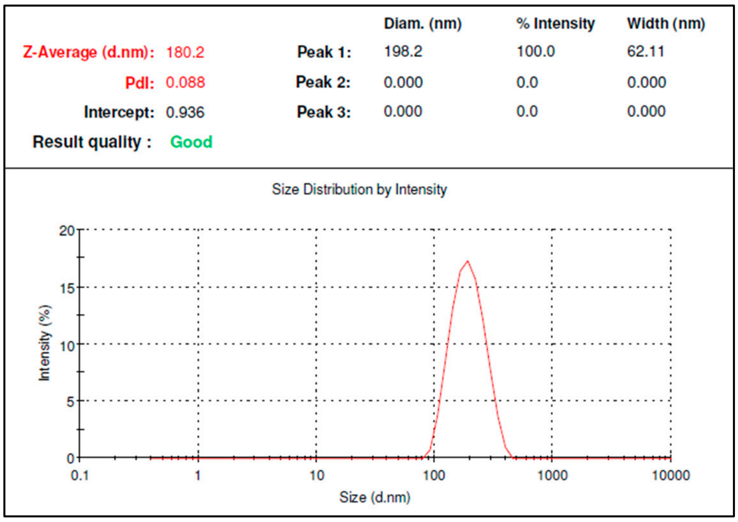

Figure S2. Representative particle size distribution curve of an ME sample.
